# Analysis of Alveolar Bone Morphology of the Maxillary Central and Lateral Incisors with Normal Occlusion

**DOI:** 10.3390/medicina55090565

**Published:** 2019-09-03

**Authors:** Ji-Eun Lee, Chang Yoon Jung, Yoonji Kim, Yoon-Ah Kook, Youngkyung Ko, Jun-Beom Park

**Affiliations:** 1 Department of Periodontics, College of Medicine, The Catholic University of Korea, Seoul 06591, Korea; 2 Department of Orthodontics, College of Medicine, The Catholic University of Korea, Seoul 06591, Korea

**Keywords:** alveolar bone loss, cone-beam computed tomography, dental implants, incisor

## Abstract

*Background and objectives:* This study investigated the morphology of the labial and palatal bony wall of the maxillary central and lateral incisors using cone-beam computed tomography (CBCT). The difference between males and females and the measurement between right and left sides were measured. *Materials and Methods:* Twenty participants, consisting of 11 females and 9 males having normal occlusion, were used for the analysis. The mean age was 21.9 ± 3.0 years. The thickness of the labial bony wall and palatal bony wall, perpendicular to the long axis of the root, were evaluated at 3 and 5 mm apical from the cemento-enamel junction (CEJ) and at the root apex. The available bony wall below the apex of the central and lateral incisors, and the angulation between the long axis of the tested tooth and outer surface of the labial bone were measured. *Results:* The mean labial bony wall thickness at the 3 and 5 mm apical from the CEJ were 1.1 ± 0.3 mm and 1.0 ± 0.4 mm for central incisors, respectively, as well as 1.2 ± 0.4 mm and 1.0 ± 0.4 mm for lateral incisors, respectively. The mean palatal bony wall thickness at 5 mm from the CEJ was above 2 mm in the central and lateral incisors. The percentage of labial bony wall thickness 2 mm or greater at the root apex in central incisors was higher than in lateral incisors (62.5% vs. 55.0%). The percentage of palatal bony wall thickness ≥2 mm at 3 mm apical from the CEJ in the central incisors was higher than in the lateral incisors (37.5% vs. 15.0%). The results on the left and right sides did not show statistically significant differences, except in the labial and palatal bony wall thickness at 3 mm from the CEJ in the lateral incisor. Generally, no significant differences were seen between males and females, but males had a significantly higher labial bony wall thickness at 3 and 5 mm from the CEJ in the central and lateral incisors when compared with females. *Conclusions:* This study showed that a majority of the cases of Korean participants had less than 2 mm of labial bony wall thickness at 3 and 5 mm apical from the CEJ at central and lateral incisors, and this should be kept in mind while performing dental practices, including tooth extraction or immediate implantation in anterior regions. Preoperative analysis using CBCT may be beneficial for establishing the treatment plan.

## 1. Introduction

Cone-beam computed tomography’s (CBCT) three-dimensional culture allows for convenient evaluation of the quality and quantity of bone [1,2]. CBCT can produce accurate and high-resolution multiple planar reformatted images with the exposure of relatively low radiation [3]. CBCT can be used as an aid during diagnosis and treatment planning, and it can minimize complications during dental operations, including tooth extraction and dental implantation [1,4]. Moreover, CBCT can be applied for the analysis of distance from anatomical structures [2]. It was shown that significant subjective benefits were achieved for additional use of CBCT in the anterior and posterior maxilla [5].

CBCT is widely used for the evaluation of bone thickness and volume of the oral and maxillofacial regions [6]. It was shown that the thickness of the crestal bone was greatest in the mandibular posterior region, followed by the mandibular anterior region and maxillary anterior region, and it was the thinnest in the maxillary posterior region [7]. Alveolar width increased from the coronal to apical direction for the central and lateral incisors, and the central incisor had significantly larger alveolar width when compared with the lateral incisor [3]. The previous report indicated that bone thickness at the apex of the maxillary incisor was greater on the palatal side when compared with the labial side in both individuals with normal occlusion and surgical skeletal Class III occlusion [8]. In another report, the thickness of the alveolar bone of maxillary central incisors with various inclinations was investigated, and it was shown that normal maxillary central incisors had a greater bony wall at the level of the root apex when compared with lingually-inclined maxillary central incisors, and that normal central incisors had a lower frequency of alveolar bone defects [9]. The previous report showed that alveolar thickness of the central incisor was greater and that the palatal cortex of the central incisor was higher than the lateral incisors in individuals, irrespective of having a long face or short face [10]. Limited information was available for the alveolar bony wall thickness with normal occlusion, and results were gathered from the maxillary and mandibular canine and premolar regions [11,12]. This study investigated the labial and palatal bony wall thickness of the maxillary central and lateral incisors using CBCT. The difference between males and females and the measurement between right and left sides were measured.

## 2. Experimental Section

### 2.1. Participants

Twenty participants, consisting of 11 Korean females and 9 Korean males having normal occlusion, were used for the analysis. The mean age was 21.9 ± 3.0 years. The Institutional Review Board at Seoul St Mary’s Hospital, The Catholic University of Korea reviewed and approved the present work (KC11RISI0585 and KC18RESI0576, 1 October 2018), and all of the experimental schemes used were performed according to relevant guidelines. The participants had dentition with periodontal health and normal occlusion. Normal occlusion was defined as follows: (1) fully developed permanent dentition having Angle Class I occlusion with normal overbite and overjet ranging from 1 mm to 3 mm; (2) spacing less than 1 mm; (3) crowding less than 3 mm; (4) no decayed or missing teeth; (5) no crown prosthesis; (6) and no facial asymmetry with crossbite.

### 2.2. Image Processing

CBCT images were taken with a 200 mm × 179 mm field of view, 80 kVp, 50 mA, resulting in a voxel size of 0.39 mm. The obtained data were exported into DICOM format and were evaluated using Invivo software (Anatomage, San Jose, CA, USA). Sagittal slices were made at the slice line through the center of the root of the maxillary central and lateral incisors.

### 2.3. Measurements

**Figure 1:**
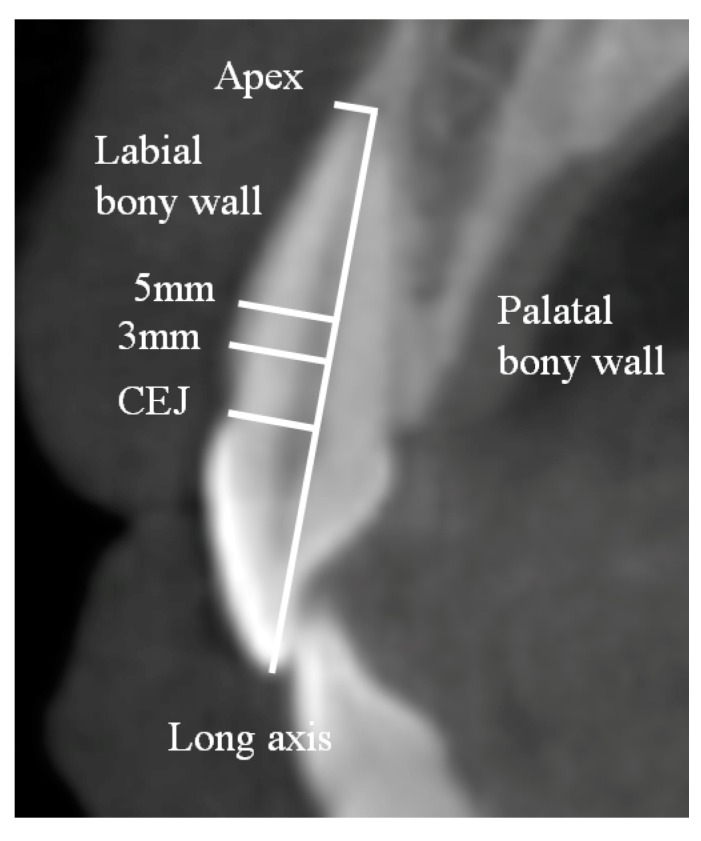
Schematic diagram measuring labial bony wall thickness of the central incisor at 3 and 5 mm from the cemento-enamel junction (CEJ) and at the root apex. The long axis was made from the incisal edge to the root apex.

The long axis was derived by connecting the tip of the cusp and the root apex in the central incisors and lateral incisors. The cemento-enamel junction (CEJ) line was defined as the line connecting the labial CEJ to the lingual CEJ. The reference point was derived by the intersection between the CEJ line and the long axis. The thickness of the labial and palatal bone was evaluated at 3 and 5 mm apical from the reference point and at the root apex (Figure 1). The distance between the root apex and apical bone was measured. Angulation between the long axis and the labial bony plate was analyzed.

### 2.4. Statistical Analysis

Tests of normality were done to determine if a data set is adequately modeled by a normal distribution. One-way analysis of variance was performed to evaluate the differences between 3 and 5 mm from the CEJ and root apex with post hoc Tukey’s test. An independent samples *t*-test was used to assess the difference in variables between males and females. A paired samples *t*-test was applied to compare the left- and right-side measurements. Commercially available statistical software was used for the statistical analysis (SPSS 12.0, SPSS Inc., Chicago, IL, USA). Statistical significance was set at a *p*-value of 0.05.

## 3. Results

Table 1 shows the thickness of the alveolar bony wall on the labial side at 3 and 5 mm apical from the CEJ and at the root apex, as well as the distance between the root tip and the apical bone. At the central and lateral incisors, the mean labial bony wall at the 3 and 5 mm positions from the CEJ was less than 2 mm. The mean labial bony wall thickness at the central and lateral incisors was greater than 2 mm at the root apex. Labial bony wall thickness at the root apex was significantly higher than labial bony wall thickness at the 3 and 5 mm points in the central and lateral incisors (*p* < 0.05). Table 2 shows the palatal bony wall thickness at 3 and 5 mm apical from the CEJ and at the root apex. In contrast to the labial area, the mean palatal bony wall thickness at 5 mm from the CEJ was greater than 2 mm in the central and lateral incisors. Palatal bony wall thickness was significantly higher at the root apex than at 3 and 5 mm from the CEJ (*p* < 0.05). A gradual increase of palatal bony wall thickness was seen from the coronal to apical direction in central and lateral incisors. The palatal bony wall thickness at 3 and 5 mm from the CEJ, and at the root apex was greater in central incisors when compared with lateral incisors, respectively (*p* < 0.05).

Table 3 presents the distribution of labial bony wall thickness at 3 and 5 mm from the CEJ and at the apex, categorized by lower than 1 mm, 1 mm or greater, and 2 mm or greater. The percentage of the thick labial bony wall (≥2 mm) at the root apex in the central incisors was greater than the lateral incisors (62.5% vs. 55.0%). Table 4 shows the distribution of palatal bony wall thickness at 3 mm and 5 mm from the CEJ and at the apex (x < 1 mm, 1 mm ≤ x < 2 mm, and x ≥ 2 mm). The percentage of the thick palatal bony wall (≥2 mm) at 3 mm from the CEJ was higher in the central incisor (37.5%) than the lateral incisor (15.0%). Similarly, the frequency distribution of the thick palatal bony wall (≥2 mm) at 5 mm from the CEJ was higher in the central incisor (77.5%) when compared with the lateral incisor (65.0%).

Table 5 presents the classification of bony wall thickness by the right and left sides. The results on the left and right sides did not produce statistically significant differences, except in the labial and palatal bony wall thickness at 3 mm from the CEJ in the lateral incisor. Table 6 shows the classification of bony wall thickness by gender. Generally, no significant differences were seen between males and females, but males had a significantly higher labial bony wall thickness at 3 mm from the CEJ and at 5 mm from the CEJ in central and lateral incisors when compared with females.

## 4. Discussion

This study demonstrated the thickness of labial and palatal bony wall at 3 mm and 5 mm apical from the CEJ in participants with normal occlusion and the percentage of the thick bony wall (≥2 mm). This study showed that in the majority of cases, the thickness of the labial bony wall was less than 2 mm in maxillary central and lateral incisors.

Previous reports have been performed to evaluate the bone thickness of maxillary anterior teeth [2,13,14,15]. In a majority of cases, the thickness of the labial bony wall of the maxillary anterior teeth is thin [13]. In another report, the average crestal bone thickness was 0.82 mm for the anterior maxilla [2]. The mean bony wall thickness at 2 mm from the CEJ of the maxillary right central and left central incisors was 0.63 ± 0.69 mm and 0.59 ± 0.71 mm, respectively, and the value for the right and left lateral maxillary incisors was 0.64 ± 0.81 mm and 0.61 ± 0.7 mm, respectively [14]. The majority of the evaluated teeth had labial bone thickness less than 1 mm at 4 mm from the CEJ (62.9%) and the middle of the root (80.1%) [4]. Similarly, 83% and 92% of the anterior tooth had labial bone thickness 1 mm or less at the crest and middle of the root, respectively [15]. The average bone thickness at the coronal, middle, and apical thirds of the labial side of the roots was 0.73, 0.69, and 0.60 mm for the central incisor, respectively, and 0.70, 0.61, and 0.49 mm for the lateral incisors [13]. The thickness of the palatal bony wall was significantly larger than the labial bony wall [13]. Most palatal thickness at the crest was thin (<1 mm, 63%), and most palatal thickness at the middle of the root and apex was thick (≥2 mm) (98%, and 99%, respectively) [15].

Use of CBCT may be helpful for immediate implantation, especially when bone augmentation is necessary [13]. It is noted that peri-implant tissue stability may be determined by the crestal bone thickness [16]. It is suggested that a 1 to 2 mm thick labial plate is necessary in immediate implant placement [17]. In another report, presurgical thickness of the labial bone was categorized as 0–0.5, 0.5–1, and ≥1 mm, and the esthetics of immediate implant placement was evaluated at 1 year follow-up; the results showed that more massive bone resorption and gingival recession were seen in groups with less than 0.5 mm thickness [17]. In the case of thin labial bone, bone augmentation seems to be recommended [18]. In a previous report, alloplastic β-tricalcium phosphate was applied with a resorbable collagen barrier membrane for bone augmentation in immediate implant placement [19]. Moreover, graft material was inserted within the gap between the implant surface and the bony wall [20]. A combinational approach with a connective tissue graft and guided bone regeneration was performed at the convexity area of the labial surface in the implant site [21,22]. Socket shield technique can be applied as an alternative for avoidance of buccal bone loss at immediate implant placement [23].

In this report, the overall measurements did not show statistical differences between the right and left sides. In a previous report, sex may have been an influencing factor [18]. This study showed that thicker labial bone was noted in males when compared with females. A previous report showed that age may also be an influencing factor [18,24]. It is reported that the labial bone thickness at the cervical portion decreases with age [18]; similarly, significantly thinner labial bone was seen in postmenopausal women [24].

Selecting the reference point seems important for evaluating the labial and palatal bony wall thickness [12]. The labial bone crest was considered a reference point in some cases [15]. It was shown that the average distance from the CEJ to the mid-labial bone crest was 2.16 mm [13]. Measurements were made at 1 mm apical, 3 mm apical, and 5-mm apical, or 1 to 5 mm apical to the labial bone crest [25,26]. CEJ was used as a reference in other studies with regard to 2 or 4 mm apical to CEJ [4,14]. For more apical regions, the middle of the root, the apex, and 4 mm beyond the apex were used [15]. In this study, labial and palatal thickness were measured at 3 and 5 mm apical to the CEJ. In this study, normal occlusion participants were used for the evaluation. In one report, anterior maxillary arches were classified as long narrow, short medium, and long medium, and the greatest bone thickness was noted in the long wide arches, followed by the long medium, short medium, and long narrow arches [27]. In another report, the position and inclination of teeth within the alveolus were used for the classification, and Classes I, II, and III indicated middle of alveolus, retroclined, and proclined teeth, respectively [15].

There are other considerations during dental procedures. Damage to the branches of the anterior superior alveolar nerve and vessels during the dental implantation may be prevented or decreased with preoperative CBCT imaging [28]. A previous report showed that the angulation between the long axis of the tested tooth and the midline of the ridge was higher with thinner labial bone thickness [29]. The thickness of the labial bony wall was positively correlated with the thickness of gingival at the level of the CEJ [30]. However, another report showed that the association between bone thickness and labial soft thickness was noted only in maxillary central incisors and not in lateral incisors [31]. Bone density can be measured using CBCT, and improvement in quality of CBCT may allow the clinicians to evaluate the trabecular bone patterns [32,33].

Computer-guided implant placement in anterior regions can be done with CBCT [34]. It should be noted that flapless immediate implant placement resulted in superior accuracy when compared with freehand surgery in transferring the implant position in the anterior maxilla using preoperative CBCT [35,36]. The average linear deviation was 0.46 mm for the implant shoulder, and the deviation was 0.67 mm for the implant apex, with an average angular deviation of 1.40 degrees [34]. The final implant position was shifted toward the labial direction when compared with the initial planning [35]. Implants with a narrower diameter when compared with the diameter of the extracted socket can be considered [20]. It was mentioned that one-fourth of the cases were not ideal for flapless immediate implant placement because of the missing labial bony wall [18]. However, underestimation of actual measurements may occur from the CBCT readings [37]. It should be also noted that bone thickness evaluation is also useful for orthodontic treatment [38,39]. It was suggested that preventive or interceptive bone augmentation may be considered for the participants receiving orthodontic treatment [39].

## 5. Conclusions

This study showed that a majority of the cases of Korean participants had less than 2 mm of labial bone thickness at 3 and 5 mm apical to CEJ at central and lateral incisors, and this should be kept in mind when performing dental practices, including tooth extraction or immediate implantation in anterior regions. Preoperative analysis using CBCT may be beneficial for establishing the treatment plan.

## Figures and Tables

**Table 1:** Labial bony wall thickness (mm) at 3 and 5 mm from the CEJ and at the root apex, including the distance from root tip to apical bone.

Parameter	Central Incisor					Lateral Incisor				
	CEJ 3 mm	CEJ 5 mm	Root Apex	Root Tip-Apical Bone	Angulation	CEJ 3 mm	CEJ 5 mm	Root Apex	Root Tip-Apical Bone	Angulation
Mean	1.1	1.0	2.3 *,**	10.4	12.4	1.2	1.0	2.2 #,##	10.5	13.3
Median	1.2	1.0	2.3	9.6	12.1	1.1	1.0	2.1	10.3	13.1
Maximum	1.9	2.0	3.9	17.5	16.7	1.9	1.9	4.4	16.1	16.0
Minimum	0.3	0.1	0.8	7.5	8.0	0.3	0.4	0.6	5.5	10.7
SD	0.3	0.4	0.8	2.7	1.9	0.4	0.4	0.8	2.2	1.3

CEJ: cemento-enamel junction. *: Significant differences were noted when comparisons were made with the 3 mm group in the central incisor (*p* < 0.05). **: There were statistical significant differences when comparisons were made with the 5 mm groups in the central incisor (*p* < 0.05). #: Significant differences were noted when comparisons were made with the 3 mm group in the lateral incisor (*p* < 0.05). ##: There were significant increases when comparisons were made with the 5 mm group in the lateral incisor (*p* < 0.05).

**Table 2:** Palatal bony wall thickness (mm) at 3 and 5 mm from the CEJ and at the root apex.

Parameter	Central Incisor			Lateral Incisor		
	CEJ 3 mm	CEJ 5 mm	Root apex	CEJ 3 mm	CEJ 5 mm	Root apex
Mean	1.9	2.7 *	7.5 *,**	1.5	2.2 #	6.1 #,##
Median	1.8	2.7	7.3	1.5	2.1	6.1
Maximum	3.6	4.4	11.2	3.1	3.9	11.1
Minimum	0.6	1.4	4.7	0.6	0.5	2.1
SD	0.6	0.7	1.7	0.5	0.7	1.7

CEJ: cemento-enamel junction. *: Significant differences were noted when comparisons were made with the 3 mm group in the central incisor (*p* < 0.05). **: There were significant differences when comparisons were made with the 5 mm groups in the central incisor (*p* < 0.05). #: Significant differences were noted when comparisons were made with the 3 mm group in the central incisor (*p* < 0.05). ##: There were significant increases when comparisons were made with the 5 mm group in the central incisor (*p* < 0.05).

**Table 3:** Frequency distribution (%) of labial bony wall thickness.

Tooth	Location	Thickness < 1 mm	1 mm ≤ Thickness < 2 mm	Thickness ≥ 2 mm
Central incisor	CEJ 3 mm	32.5	67.5	0.0
	CEJ 5 mm	45.0	52.5	2.5
	Root apex	2.5	35.0	62.5
Lateral incisor	CEJ 3 mm	32.5	67.5	0.0
	CEJ 5 mm	47.5	52.5	0.0
	Root apex	2.5	42.5	55.0

CEJ: cemento-enamel junction.

**Table 4:** Frequency distribution (%) of palatal bony wall thickness.

Tooth	Location	Thickness < 1 mm	1 mm ≤ Thickness < 2 mm	Thickness ≥ 2 mm
Central incisor	CEJ 3 mm	5.0	57.5	37.5
	CEJ 5 mm	0.0	22.5	77.5
	Root apex	0.0	0.0	100.0
Lateral incisor	CEJ 3 mm	10	75	15
	CEJ 5 mm	2.5	32.5	65
	Root apex	0	0	100

CEJ: cemento-enamel junction.

**Table 5:** Classification of bony wall thickness by topology (right and left side).

Tooth	Topology	Labial					Palatal		
		CEJ 3 mm	CEJ 5 mm	Apex	Root Tip-Apical Bone	Angulation	CEJ 3 mm	CEJ 5 mm	Apex
Central incisor	Right	1.1 ± 0.4	1.0 ± 0.4	2.3 ± 0.8	10.5 ± 2.3	12.4 ± 1.7	2.0 ± 0.7	2.7 ± 0.8	7.5 ± 1.8
	Left	1.1 ± 0.3	1.0 ± 0.4	2.3 ± 0.8	10.3 ± 2.7	12.4 ± 2.1	1.8 ± 0.5	2.6 ± 0.7	7.5 ± 1.7
	*p*-value	>0.05	>0.05	>0.05	>0.05	>0.05	>0.05	>0.05	>0.05
Lateral incisor	Right	1.3 ± 0.3	1.1 ± 0.4	2.1 ± 0.7	10.2 ± 2.1	13.8 ± 1.2	1.7 ± 0.5	2.4 ± 0.7	6.1 ± 1.5
	Left	1.0 ± 0.4 *	1.0 ± 0.4	2.2 ± 0.8	10.9 ± 2.3	12.8 ± 1.2	1.4 ± 0.4 *	2.1 ± 0.7	6.0 ± 1.9
	*p*-value	0.000	>0.05	>0.05	>0.05	0.007	0.019	0.094	0.838

CEJ: cemento-enamel junction. *: There were statistically significant differences when comparisons were made with the right side in each group.

**Table 6:** Classification of bony wall thickness by gender.

Tooth	Gender	Labial					Palatal		
		CEJ 3 mm	CEJ 5 mm	Apex	Root Tip-Apical Bone	Angulation	CEJ 3 mm	CEJ 5 mm	Apex
Central incisor	Male	1.3 ± 0.3	1.1 ± 0.4	2.5 ± 0.9	10.7 ± 3.1	12.7 ± 2.2	2.1 ± 0.7	2.8 ± 0.8	7.8 ± 1.4
	Female	1.0 ± 0.3 *	0.9 ± 0.4 *	2.2 ± 0.6	10.2 ± 2.4	12.2 ± 1.62	1.7 ± 0.5	2.5 ± 0.7	7.2 ± 1.9
	*p*-value	0.000	0.045	>0.05	>0.05	>0.05	>0.05	>0.05	>0.05
Lateral incisor	Male	1.4 ± 0.4	1.2 ± 0.3	2.4 ± 0.9	10.8 ± 2.6	13.4 ± 21.4	1.6 ± 0.5	2.2 ± 0.6	7.0 ± 1.4
	Female	1.0 ± 0.3 *	0.9 ± 0.3 *	2.0 ± 0.6	10.3 ± 1.9	13.2 ± 1.2	1.5 ± 0.4	2.2 ± 0.8	5.3 ± 1.6 *
	*p*-value	0.002	0.001	>0.05	>0.05	>0.05	>0.05	>0.05	0.002

CEJ: cemento-enamel junction *: Statistically significant differences were noted when comparisons were made with the males in each group.
